# Influence of Process Parameters on the Kinetics of the Micelle-to-Vesicle Transition and Ripening of Polystyrene-Block-Polyacrylic Acid

**DOI:** 10.3390/polym15071695

**Published:** 2023-03-29

**Authors:** Jil Mann, Julian K. Mayer, Georg Garnweitner, Carsten Schilde

**Affiliations:** 1 Institute for Particle Technology, Technische Universität Braunschweig, Volkmaroder Str. 5, 38104 Braunschweig, Germany; 2 Laboratory for Emerging Nanometrology, Technische Universität Braunschweig, Langer Kamp 6A, 38106 Braunschweig, Germany; 3 Battery LabFactory, Technische Universität Braunschweig, Langer Kamp 8, 38106 Braunschweig, Germany

**Keywords:** block copolymer, self-assembly, kinetics, morphology, micelle, cylinder, vesicle, transition, ripening, process engineering

## Abstract

Due to their ability to self-assemble into complex structures, block copolymers are of great interest for use in a wide range of future applications, such as self-healing materials. Therefore, it is important to understand the mechanisms of their structure formation. In particular, the process engineering of the formation and transition of the polymer structures is required for ensuring reproducibility and scalability, but this has received little attention in the literature. In this article, the influence of the addition rate of the selective solvent on the homogeneity of self-assembled vesicles of polystyrene-block-polyacrylic acid is demonstrated, as well as the influence of the reaction time and the mixing intensity on the morphology of the polymer structures. For example, it was demonstrated that the higher the mixing intensity, the faster the transition from micelle to vesicle. The experimental results are further supported by CFD simulations, which visually and graphically show an increase in shear rate and narrower shear rate distributions at higher stirring rates. Furthermore, it was demonstrated that the vesicle size is not only kinetically determined, since flow forces above a critical size lead to the deformation and fission of the vesicles.

## 1. Introduction

An example of self-organizing systems from nature is the assembly of phospholipids to form our cell membrane [1]. The principle is based on a non-covalent association of amphiphilic molecules, which enables a high nanoscale structural precision, as it is mandatory, for example, in the development of functional nanosystems [2,3]. To transfer this principle, an in-depth understanding of the thermodynamics and kinetics of self-assembly processes is essential. For this purpose, block copolymers are very suitable as model amphiphiles, since their molecular weights are very high compared to low-molecular weight surfactants. As a result, phase transitions take place much more slowly, so that the polymer structures could be investigated more comprehensively [4].

A very common method for triggering self-organization is the co-solvent method. First, the block copolymer is dissolved in a solvent (usually organic) that has a high affinity for both the hydrophilic and the hydrophobic part of the amphiphile. By adding a selective solvent such as water, the block copolymers assemble into mesoscopic structures above a critical amount of solvent (critical water content, or cwc) with the aim of generating the smallest possible interface. This method is also called microphase separation because it is characterized by the precipitation of the insoluble part of the block copolymer (usually the hydrophobic part), while the other part (usually the hydrophilic part) remains in solution [1]. The ratio of the volume of the hydrophobic part v to the contact area of the hydrophilic part a_0_ and the length of the hydrophobic part l_c_ of the block copolymer determines which associate structure will be formed. This ratio, which was first described in 1976 by Israelachvili et al. [5], is known as the so-called packing parameter *p*, and this is defined by the following equation (Equation (1)):

*p* ₌ v/(a_0_ × l_c_) (1)

A polymer molecule showing *p* ≤ 1/3 tends to form spherical micelles, a molecule with *p* ≥ 1/3 and *p* ≤ 1/2 cylindrical micelles, and a molecule with *p* ≥ 1/2 and *p* ≤ 1 lamellae or vesicles. If the packing parameter is greater than 1, the above-mentioned structures are formed in the opposite order as inverse structures [1]. The packing parameter of a polymer molecule, and thus, the morphology of the block copolymer structure, should, however, by no means be regarded as fixed. Different authors have described, the effects of the type and amount of solvent and antisolvent [6,7], the composition of the block copolymer [8,9], and the concentration of the polymer, as well as the presence of additives [9]. 

In addition to the above-mentioned structures, much more complex morphologies have been observed, for example, as metastable transition morphologies [10]. Therefore, many different and partly complex morphologies have been obtained from block copolymers, such as spherical or cylindrical micelles [7,8,11], disc-like micelles [12,13], toroids [14], spherical particles with gated nanopores [15], vesicles (e.g., genus vesicles, multi-compartment vesicles, Janus vesicles, and onion-like vesicles) [16], lamellar structures, network-like structures [7,11], helical structures [17], and many more [18] could be revealed so far. This diversity results in many potential applications, such as in the fields of nanocomposites [7,19], biomaterials [20], drug delivery systems [21,22,23,24], nanoreactors [25], or as self-healing materials [26].

In recent years, some research has been performed on the pathways of the morphological transitions from one polymer structure to the other. Amongst others, the transitions from micelles to vesicles [12,27,28], micelles to cylinders [4,28,29], cylinders to disk-like micelles [12], cylinders to donuts [14,30], and cylinders to vesicles [31,32], were investigated. Despite the great interest, the complete mechanism of the structure transitions has not yet been completely clarified. A previously well-accepted but rather simple view assumes that the vesicles form via a two-step process. First, a bilayer membrane is formed from the polymer chains, which bends and closes in the next step to form a hollow structure. In reality, however, the occurring processes are believed to be much more complicated [33]. Leng et al. [12] demonstrated that micelles (spherical and cylindrical) transform into disk-like intermediate micelles in a first, very fast step (within 1 s). Subsequently, growth occurs through contact and coalescence between disk-like micelles up to a critical radius r*. Once this critical size has been reached, the vesicles are finally formed through the closing of the structures. The transition morphology is reminiscent of a basket. The subsequent ripening of the vesicles could not be observed directly, but was expected if there was enough time to reach equilibrium. A very similar mechanism was found by Rank et al. [34]. Burke and Eisenberg [4], observed that cylindrical micelles were formed from spherical micelles. This was followed by a rapid adhesive collision of micelles to “pearl necklace” structures, and their subsequent reorganization to smooth cylindrical micelles. Furthermore, in addition to the theory of disk-like structures as the transition morphology between cylinders and vesicles, the formation of donuts from cylinders is also currently being discussed. It is postulated that there is a curvature and a subsequent linking of one end to the other end of the cylinder [30,35,36]. 

However, to our knowledge, there is currently no literature that has investigated the influence of the process parameters studied here on the micelle-to-vesicle transitions from block copolymers. So far, the temperature has been a well-studied process parameter [37,38,39,40]. For example, Sundararaman et al. [38] demonstrated that small spherical micelles of the triblock copolymer PEO-b-PNIPAM-b-PI (poly(ethylene oxide)-b-PNIPAM-b-poly(isoprene)) existed in 20 °C warm water, which could be converted to vesicles when the water temperature was increased to 65 °C. 

Therefore, the aim of this work was to improve the knowledge by investigating the effects of important process parameters on the intra- and interparticle interactions of mesoscopic block copolymer structures, allowing for a deeper understanding of the structure formation and transformation processes. Therefore, we want to highlight the influence of the rate of the addition of the selective solvent to the polymer solution, stirring speed, reaction time (the time between the last addition of the selective solvent and the freezing of the current state (referred to herein as the structure formation time)) on the morphology and the dimension of the polymer structures, and thereby derive structure formation mechanisms from micelle to vesicle. In addition, the CFD simulations of the flows in the piston during the experiment will support our results and assumptions.

## 2. Materials and Methods

### 2.1. Materials

Polystyrene-block-polyacrylic acid (PS-b-PAA, batch: MKBQ5839V) was purchased from Sigma Aldrich (Steinheim, Germany). The degree of polymerization of PS is 275 and of PAA 30, as stated by the manufacturer. The molecular weights (M_n_) and the polydispersity index (PDI) of PS-b-PAA are summarized in Table 1.

Deionized water was used as the selective solvent for the hydrophobic part (polystyrene). Tetrahydrofuran (THF, for HPLC, not stabilized) and 1,4-dioxane (for HPLC, not stabilized) from Carl Roth (Karlsruhe, Germany) were utilized as good solvents for PS-b-PAA.

Filters were used for microscopy (Whatman^®^ Nuclepore™ track-etched polycarbonate membranes, with pore diameters of between 0.2 µm and 2.0 µm, Dassel, Germany). A quartz glass cuvette (Hellma Analytics, Type 100 QS, Müllheim, Germany) with a layer thickness of 10 mm and a nominal volume of 3.5 mL was used for dynamic light scattering measurements.

### 2.2. Preparation of Polymer Structures of PS-b-PAA

**Figure 1:**
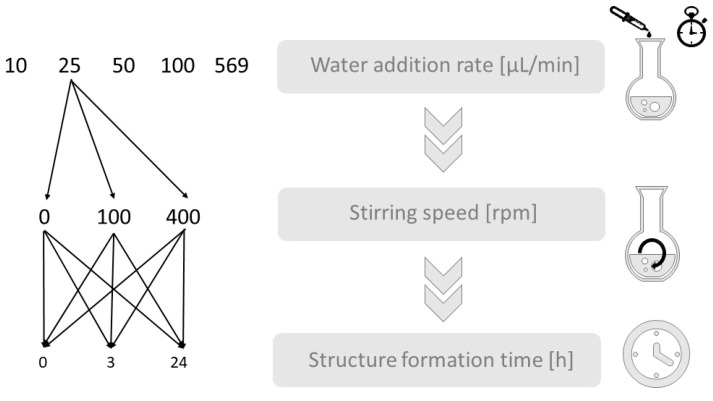
Schematic diagram of the experimental procedure to investigate the influence of the process parameters for water addition rate, stirring speed, and structure formation time on the morphologies of polystyrene-block-polyacrylic acid polymer structures.

For the preparation of the polymer structures, the co-solvent method was used. For this, polymer solutions with PS-b-PAA were prepared in THF or dioxane at an initial concentration of 3 mg/mL, and completely dissolved overnight with stirring. The polymer solution was then placed in a 25 mL round bottom flask, diluted with THF or dioxane, and the flask made airtight with septa. To prevent solvent loss due to evaporation during the experiments, the flasks were kept tightly closed for the entire duration and only opened for a short moment, e.g., to take a sample or to add a selective solvent. Consequently, no changes in volume due to evaporation losses were observed. The polymer solutions were then stirred at high speed (400 rpm) for 15 min after dilution to ensure adequate mixing. Water was subsequently added as a selective solvent at a fixed ratio and stirring speed, according to the test conditions of the parameter matrix (see Figure 1). For this purpose, a 2–200 µL Eppendorf pipette was used, and the timing of the addition was ensured using a stopwatch. An accuracy of ±5 s was maintained. After the addition of the respective amount of water was completed, the system was given a defined time to form the aggregates (which we refer to as the structure formation time). For all experiments, the same water content of 14 wt%, a polymer concentration of 0.1 wt%, and a total volume of 5 mL were set.

The experimental setup was utilized to investigate the influences of the process parameters of water addition rate, stirring speed, and structure formation time on the morphology of the resulting block copolymer structures, schematically shown in Figure 1. Accordingly, the water addition rate was first examined in more detail. It varied from 10 µL/min, 25 µL/min, 50 µL/min, and 100 µL/min, to 569 µL/min (569 µL corresponds to exactly 14 wt% water, so this amount was added all at once and then stirred for exactly one minute) using the solvent THF and a stirring speed of 400 rpm during the water addition, and of 100 rpm during the 3-h structure formation time. Then, a water addition rate of 25 µL/min was fixed for the subsequent investigation of the influence of the stirring speed and the structure formation time. For each stirring speed (0, 100, and 400 rpm), three different structure formation times (0, 3, and 24 h) were investigated.

**Figure 2:**
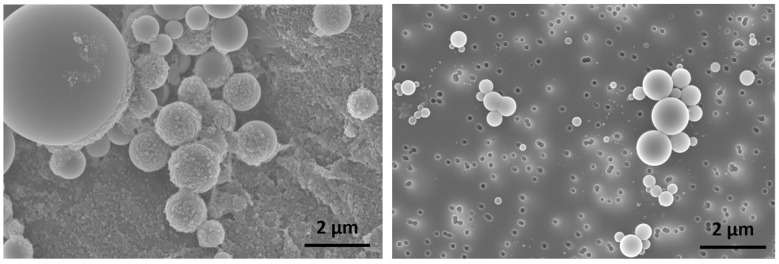
Exemplary SEM images of PS-b-PAA vesicles (prepared via the co-solvent method with 16.2 wt% water, and a polymer concentration of 0.05 wt% in the solvent THF) without centrifugation (**left**) and with centrifugation (**right**), to study the purification procedure.

At the end of each experiment, water was abruptly added to the solution in a 3-fold excess to freeze the kinetics of structure formation, with a final particle concentration of 0.02 wt%. Samples were then centrifuged at 9500 rpm for 15 min and redispersed in the same amount of water to sufficiently clean and stabilize them. Preliminary studies demonstrated that this procedure does not result in any morphological changes, and that this method is well suited to purify the samples (Figure 2). Immediately after washing, the samples were prepared for SEM measurements.

### 2.3. Sample Preparation and Characterization

For the SEM (Helios G4 CX from FEI, voltage: 5 kV, current: 0.8 nA, Frankfurt am Main, Germany) sample preparation, about 50 µL to 150 µL of the respective mixture was put onto a membrane filter. Because of an underlying paper under the membrane, the solvent was extracted through the membrane pores via capillary forces. This procedure should ensure that there are no drying effects on the sample. The filter was then dried at atmospheric pressure, glued onto a copper grid, and sputtered with 4 nm of platinum.

The critical water content (cwc) was determined with dynamic light scattering (Zetasizer from Malvern, Worcestershire, UK). Three measurements per sample were carried out for the solvents THF and dioxane. The experiments were performed directly in the cuvette to minimize solvent losses.

### 2.4. Computational Fluid Dynamics (CFD)

**Figure 3:**
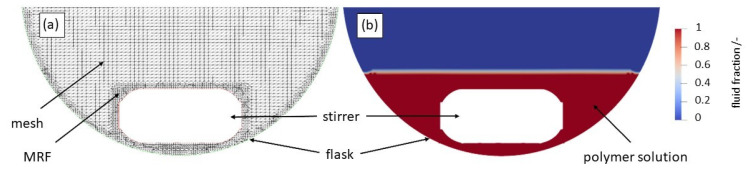
Generated mesh (**a**) and fluid phases (**b**) of the developed setup. The edges of the geometries are shown in colour (blue = piston and red = stirrer).

CFD simulations were performed to gain a deeper insight into the flow conditions and resulting shear rates during the process. Heyn et al. [41] were already able to demonstrate that this method is very well suited to generating additional information on the structure formation of organic systems, depending on the process or the flow pattern. However, the geometric dimensions of the flask and of the stirring bar were measured manually. The geometries used were prepared in the stereolithography (STL) format using SolidEdge ST9. The simulations were conducted with open-source software OpenFOAM 7 (OF). The solver interFoam was chosen for isothermal non-miscible incompressible fluids and multiphase flow with the volume of fluid (VOF) method, which includes possible mesh motions, optional topology changes, and adaptive re-meshing [42]. To simulate the rotation of the stirrer, a multiple reference frame (MRF) approach was chosen. The mesh generation was performed with the OF tools “blockMesh” for the basic mesh (75 cells in the x, y, and z directions each) and “snappyHexMesh” for the implementation of the STL geometries (flask and stirrer), as well as the detailing and refinement of the mesh. The refined mesh has a total cell number of 362,806. A section through the created mesh can be observed in Figure 3a. A finer mesh was created around the flask walls and for the MRF around the stirrer, to account for zones of high velocities and stressing.

The regions of polymer solution and air were generated with “setFields” in such a way where the flask was filled with solution, up to a height of 10 mm. To estimate the turbulence of the flow, Reynolds numbers (using Equation (2), where ρ_fl_ is the fluid density, n the stirrer velocity, d the diameter of the stirrer, and η the dynamic viscosity of the fluid), were calculated using material data from the literature for the density and viscosity of the fluid [43]. Water at T_fl_ = 20 °C was assumed for the parameters of the solution. The stirring bar velocity was set to 10.47 (100 rpm) and 41.89 rad s^−1^ (400 rpm), respectively. The Reynolds numbers for both stirring velocities were well below the threshold of 10,000 (approx. 371 and 1483, respectively), which meant that laminar flow could be assumed for the simulation.

Re_stirring_ = (ρ_fl_ × n × d^2^)/η (2)

The basic governing equations (the conservations of mass and momentum) were calculated using the basic Navier–Stokes equations. The equations of continuity and motion were solved to attain the velocities and velocity profiles. Thereafter, shear rates were calculated for each cell and then transferred into a distribution. For each speed of the stirrer, after 10 revolutions (6 or 1.5 s, at 100 or 400 rpm), the results of the next 10 revolutions (up to 12 or 3 s, respectively) were averaged to account for the transient type simulation. Images of the setup and the flow pattern were generated using ParaView 5.6.0. For the initial conditions at the walls, a zero flux was imposed to the computational domains, and the gravity was set to 9.81 m s^−1^.

## 3. Results and Discussion

### 3.1. Determination of Critical Water Content

**Figure 4:**
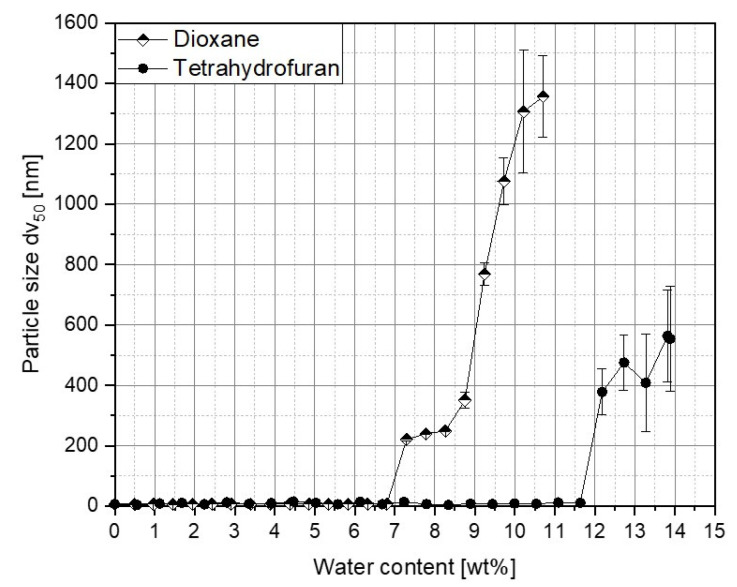
Determination of critical water contents using dynamic light scattering. Block copolymer solutions of 0.1 wt% with THF and dioxane were investigated.

In order to study the self-assembly of the block copolymers, it is first important to know at what amount of the selective solvent water the structure formation begins. For this purpose, water was successively added to 0.1 wt% PS-b-PAA solutions in THF and dioxane, and the particle size was measured. A significant rise in particle size, and therefore, an indication of the critical water content (cwc) was determined for the solvent THF at 12.2 wt%, and for dioxane, at 7.3 wt% (compare Figure 4).

This can be explained by the different solubilities of the polymer in the solvents. It is of great importance as to how well that part of the polymer dissolves in the solvent, which will be precipitated by the selective solvent; in this case, the solubility of polystyrene in the organic solvent. The Hansen parameter is a characteristic parameter for the prediction of the solubility. The more similar the Hansen parameters of the solvent and polystyrene, the better the solubility and the greater the amount of selective solvent that is necessary to induce microphase separation. The Hansen parameter of THF amounts to 19.46, and thus, it is more similar to the parameter of polystyrene of 19.26 than the one of dioxane with 20.47 [44]. As a result, experimentally determined differences in the critical water contents are expected.

THF was then chosen as the solvent for all further experiments because it has a slower phase transition compared to dioxane, because the solubility parameter is more similar to that of polystyrene.

### 3.2. Influence of Water Addition Rate on the Vesicle Size Distribution

**Figure 5:**
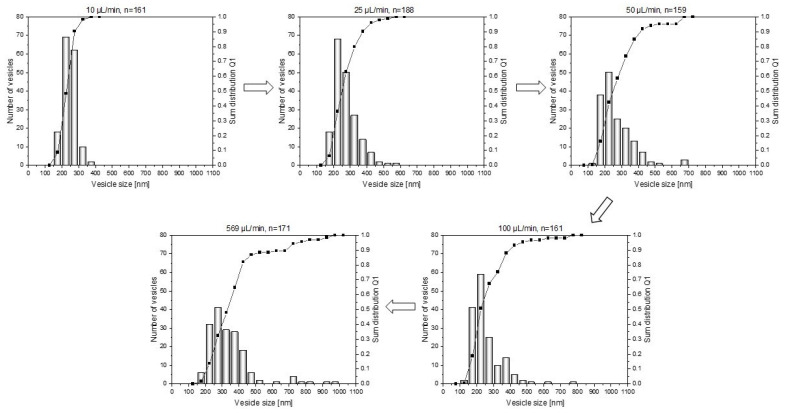
Influence of the addition rate of the selective solvent water on the vesicle size distribution (shown as number distributions (histograms) and sum distributions (line charts)) with THF as good solvent for a water content of 14 wt% and a constant stirring speed of 100 rpm.

Before the series of experiments on structure formation and transformation could be initiated, the addition rate of the selective solvent had to be determined. Since this can have an influence on the homogeneity (here, measured via the vesicle size distribution), it was necessary to examine this parameter in more detail beforehand. For this purpose, representative SEM images were used to determine the sizes and distributions of the vesicles. As expected, a trend can be observed: The faster the addition, the larger the average vesicle size, and the broader the particle size distribution (see Figure 5).

Furthermore, the average vesicle size and the polydispersity (PDI) can be taken from Table 2. The PDI was determined from the sum distribution Q_1_ (specifically, the length distribution), according to Equation (3).

PDI = (x_90_ − x_10_)/x_50_ (3)

**Figure 6:**
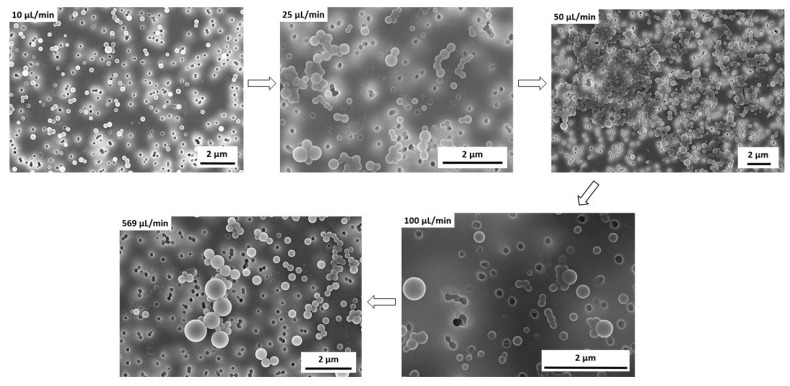
Representative SEM images in the study of the addition rate of the selective solvent water on the size distribution of PS-b-PAA vesicles, with THF as a good solvent at a water content of 14 wt% and a constant stirring speed of 100 rpm.

For further support, representative SEM images are also shown in Figure 6.

If the selective solvent is added very quickly, large differences in local supersaturation occur in the solution. As a result, in addition to vesicles that have already formed, there is also a high monomer concentration of block copolymers, which means that there is both growth of the vesicles that are already present, and in the further course of time, the formation of new vesicles. If vesicle formation and vesicle growth or ripening are strongly separated in time, inhomogeneous systems or broad particle size distributions result. If, on the other hand, the selective solvent is added slowly, it is distributed homogeneously in the flask and initiates vesicle formation at the same time, resulting in more homogeneous systems.

Furthermore, Table 2 shows that in addition to a broadening of the vesicle size distribution with a fast addition of water, the average vesicle size also tends to increase. Here, the dominant effect is assumed to be Ostwald ripening. Accordingly, the large particles continue to grow, at the expense of the small particles.

As a compromise between a short process time and an acceptable vesicle size distribution, all further experiments (the influence of mixing intensity and structure formation time) were carried out, with a water addition rate of 25 μL/min.

### 3.3. Influence of Mixing Intensity and Structure Formation Time on the Polymer Structure of PS-b-PAA

In the following, the process parameters for mixing intensity (varied via the stirring speed) and structure formation time were considered. As mentioned above, the structure formation time is defined as the time that has elapsed between the end of the addition of the selective solvent to the defined water content of 14 wt% (corresponding to the time point t = 0 h) and the stop of self-assembly, by freezing the morphologies of the polymer structures.

**Figure 7:**
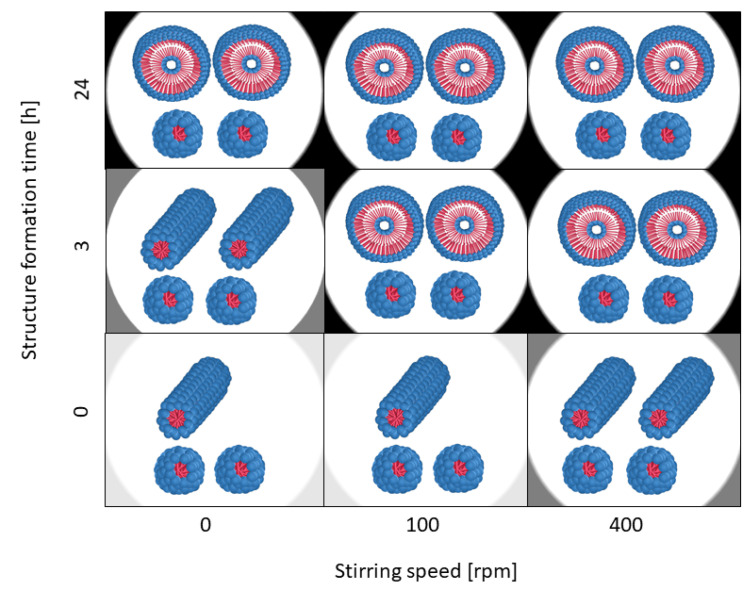
Schematic representation of the morphologies of the polymer structures (blue for the hydrophilic head PAA and red for the hydrophobic part PS), as detected from representative SEM images for each sample, as a function of the structure formation time and the stirring speed. The colored backgrounds distinguish the number and type of morphology (light grey = few cylindrical structures, medium grey = higher number of cylindrical structures and black = vesicles).

**Figure 8:**
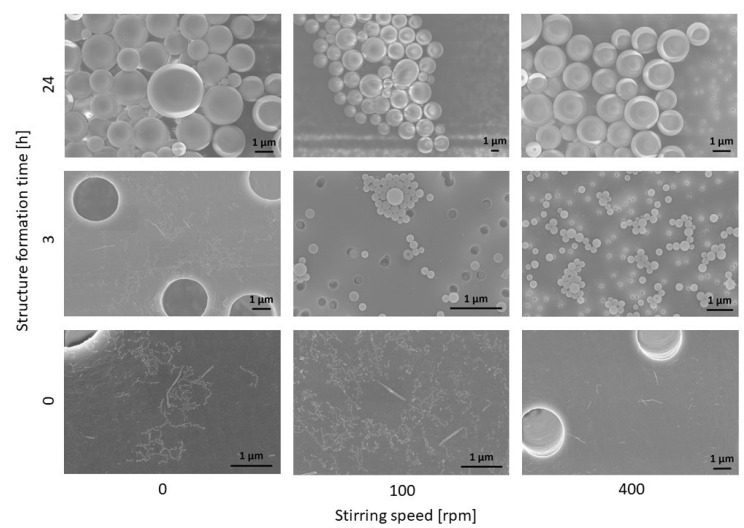
Representative SEM images of the morphologies of the polymer structures as a function of the structure formation time and the stirring speed.

Figure 7 schematically shows the morphologies of the polymer structures, and Figure 8 shows the corresponding SEM images, as a function of structure formation time and mixing intensity. At this point, it should be mentioned that in all samples, there were many micelles with particle sizes of around 30 nm. As they were observed in all samples, it is very likely that this is a co-existing phase, which amongst other things, could be due to the polydispersity of the block copolymer (see Table 1). Blocks with different lengths could result in more than one thermodynamically stable morphology in the same sample [1]. For all further considerations, this co-existing phase is not taken into account further, because it did not provide any deeper information in this study.

As it can be observed from Figure 7 and Figure 8, both the mixing intensity and the structure formation time have a significant influence on the morphology of the resulting polymer structure. If there is no mixing (0 rpm), a few cylinders and numerous micelles can be observed. If the mixing intensity increases to 400 rpm, the number of cylinders also increases. This is shown graphically in Figure 7 in such a way that the number of morphologies indicates their frequency in the sample (one cylinder = few, and two cylinders = significantly more cylindrical structures detected). However, no significant difference in the number of cylinders between 0 rpm and 100 rpm can be observed. A similar increase in the ratio of formed cylinders can be observed when increasing the structure formation time for a low mixing intensity.

**Figure 9:**
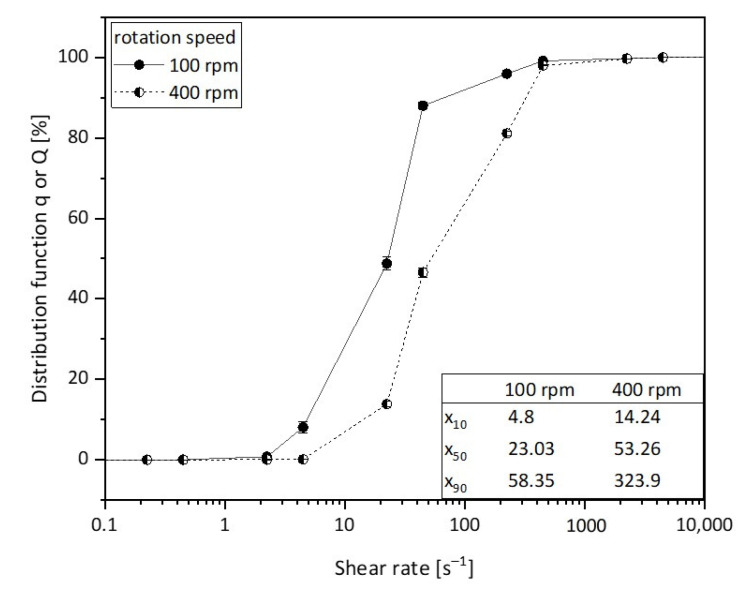
Representation of the simulated shear rate distributions of the fluids at 100 rpm and 400 rpm.

Furthermore, it can be observed that with a further rise in the mixing intensity and/or structure formation time, the cylinders disappear completely and the vesicles are detected instead. With a high probability, the latter can be regarded as the equilibrium morphology, which is why we assume that our system has a packing parameter of between 1/2 and 1, since, as known from the literature [5], vesicles are present at this packing parameter. It is obvious that the cylindrical structures reorganize into spherical structures for longer structure formation times. In summary, both time and kinetic energy (due to mixing) have a significant influence on the vesicle formation process. The higher the structure formation time and the higher the mixing intensity, the faster the formation of vesicles as the equilibrium structure occurs. We postulate that, essentially, a higher shear rate at higher rotational speeds leads to larger collision probabilities, which accelerates the structure formation mechanisms. This is also suggested by our simulative experiments. In Figure 9, the shear rate distributions of the fluids are plotted for 100 rpm and for 400 rpm. It can be observed that with an increase in speed, a higher shear rate can be obtained. This can be observed particularly significantly for x_90_. A disproportionate increase in the shear rate from 58.35 s^−1^ to 323.9 s^−1^ was determined here.

The difference can also be well observed from the simulated velocity profiles (see Figure S1).

This shows that it is absolutely necessary that the process, and not only the formulation, must be considered in phase transition processes from self-assembled block copolymers.

**Figure 10:**
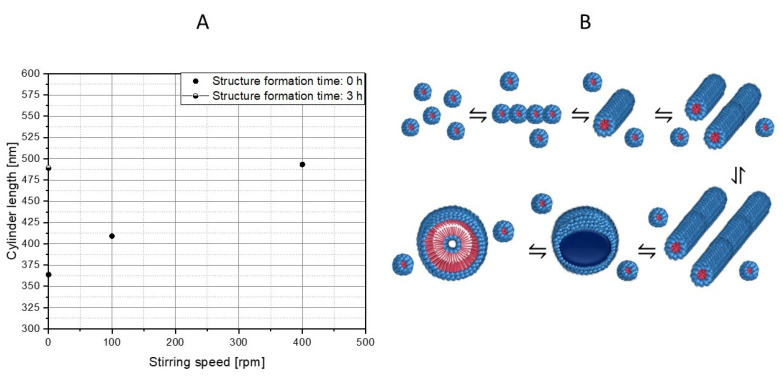
Influence of stirring speed and structure formation time on the lengths of the cylinders (**A**). Derived structure formation mechanism, from micelle formation to vesicle emergence (**B**). The hydrophilic part PAA is shown in blue and the hydrophobic part PS of the block copolymer is shown in red.

Based on the observed morphological changes, structure formation mechanisms can be derived, which only relate to the PS-b-PAA system considered here. However, based on the simulative data, which does not refer to the specific chemistry of the polymer system, a transfer to other self-assembled block copolymers of similar chain lengths seems to be possible. For this purpose, however, it makes sense to also examine dimensional changes (see Figure 10).

Regarding the dimensional change of the cylinders based on the SEM evaluation, it can be observed that the average length of the cylinders rises continuously with the increasing mixing intensity (363.7 nm ± 313.0 nm, n = 53 (0 rpm, 0 h), 409.0 nm ± 228.9 nm, n = 48 (100 rpm, 0 h), and 493.2 nm ± 583.5 nm, n = 90 (400 rpm, 0 h)). In the absence of mixing, a longer structure formation time also leads to longer cylinders (compare 0 rpm at 0 h and 3 h (489.1 nm ± 374.0 nm, n = 89)). It could also be determined that with a value of 493.2 nm, the average cylinder length at 400 rpm and 0 h corresponds approximately to the average length after 3 h at 0 rpm with 489.1 nm. It is obvious that the cylinders have a critical length that roughly corresponds to this length, since only vesicles are present in the samples as the structure formation progresses. The cylinder growth can also be attributed to higher collision probabilities because of higher shear rates. It is expected that the collision of micelles with cylinders, as well as the collision of two cylinders, leads to growth. In contrast, the growth due to individual polymer molecules in the solution will play a rather minor role, since solutions with block copolymers have a very low critical micelle concentration, and therefore, the number of monomers is likely to be low [45].

**Figure 11:**
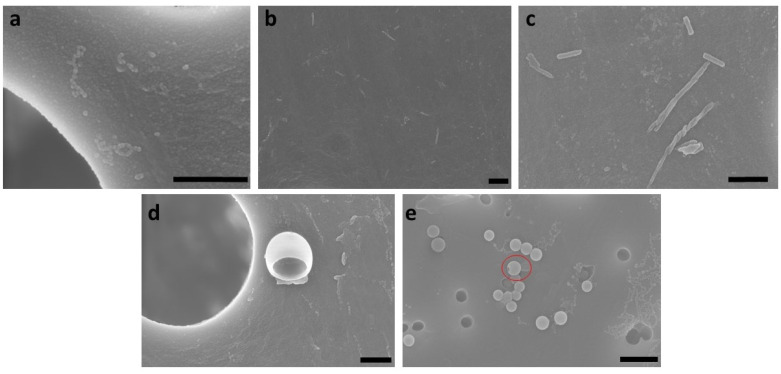
Exemplary SEM images to illustrate the postulated micelle-to-vesicle transition. (**a**) and (**b**) 0 rpm, 0 h, pearl necklaces from micelles and short smooth cylindrical micelles, (**c**–**e**) 400 rpm, 0 h, partially agglomerated, twisted cylindrical micelles (short and long), (**d**) 400 rpm, 0 h, basket structure, and (**e**) 100 rpm, 3 h, small vesicles (partly but not yet completely closed (see red circle)). The scale bar is 500 nm.

A structure formation mechanism from micelle to vesicle can be established from the SEM images (compare Figure 11). In the first step, spherical micelles are formed, which then, upon contact, initially assemble into “pearl necklaces” (Figure 11a). Burke and Eisenberg previously postulated a reorganization mechanism resulting in smooth cylindrical structures [4], and indeed, such structures can also be observed (Figure 11b), although we could not resolve the kinetics of this process, as both states occur at the same time step. When stirring at higher rates, longer cylinders form that partially become curved (Figure 11c), whilst after an extended reaction time, an increasing proportion of vesicles can be observed (Figure 11d,e). Hence, we postulate that the cylinders grow to a critical length l_c_* and ultimately form the vesicles. Additionally, we could observe basket structures as an intermediate state (a clear basket structure can be observed in Figure 11d), and vesicles with a small punctual opening on their surface (the red circle indicates the example in Figure 11e), which indicates that they were formed from the basket structures. These transitional structures suggest that previously, disk-like structures had formed as intermediates. It is conceivable that these disk structures were created through a fusion of micelles and cylinders, and then they reorganized into flat, round structures. As schematically shown in Figure 7, the number of cylinders increases with time and mixture intensity, which also increments the likelihood of contacts between the cylinders.

The key factor here is that the cylinders or disks must curve to form vesicles. The predominant driving force here is the minimization of free energy due to the open-end caps of the cylinders. On the other hand, there is the bending modulus, which has to be small enough to allow for curvature [46]. This bending module is, of course, very high for small cylinders, as compared to large cylinders. Thus, we assume that the cylinders grow to the size where the bending modulus becomes small enough to enable curling, which corresponds to the critical length l_c_*.

**Figure 12:**
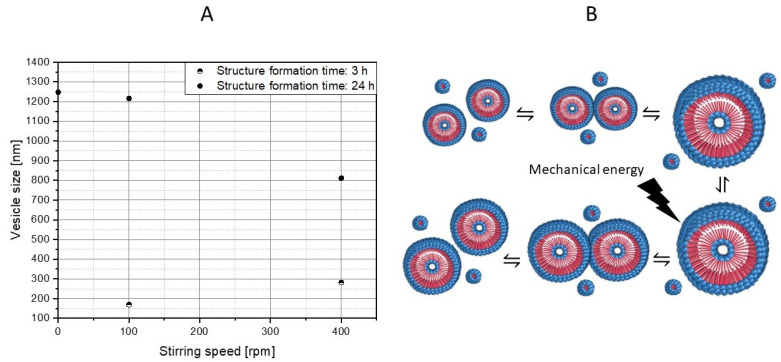
Influence of the stirring speed and the structure formation time on the vesicle sizes of the polymer structures (**A**). Derived ripening processes, as well as behavior of vesicles in the flow (**B**). The hydrophilic part PAA is shown in blue and the hydrophobic part PS of the block copolymer is shown in red.

Once the vesicles are formed, they grow until they reach kinetic equilibrium. Therefore, the vesicles continued to be observed for a total of 24 h at different mixing intensities (see Figure 12). If only the experimental series with a structure formation time of 3 h is considered, an increase in the vesicle size can be determined with the higher mixing intensity (169.4 nm ± 49.2 nm, n = 260 (100 rpm, 3 h) and 281.6 nm ± 59.1 nm, n = 225 (400 rpm, 3 h)). As has already been discussed for the cylinder growth, this is mainly a result of a higher collision probability via an increased particle movement. Likewise, a significant rise in particle size over time at the same rotation speed can be observed (compare 100 rpm at 3 h and 100 rpm at 24 h (1215.7 nm ± 563.5 nm, n = 216)), which is also attributed to a higher contact probability. If, on the other hand, the experimental series at 24 h is considered, an insightful trend can be observed. Between 0 rpm (1247.1 nm ± 863.2 nm, n = 276) and 100 rpm, there seems to be no significant difference in vesicle size. We assume that the vesicle size of the kinetic equilibrium was already reached. If, on the other hand, the mixing intensity is raised further, a significant decrease in the vesicle size can be observed (810.9 nm ± 629.1 nm, n = 240 (400 rpm, 24 h)). A possible conclusion would be that flow-induced forces become relevant at above a critical vesicle size. When the deforming stresses acting on the vesicle surface exceed the retained stresses, particle rupture occurs, or in the case of polymer vesicles, fission occurs. Accordingly, the vesicle size of the kinetic equilibrium would also depend on the prevailing flow conditions.

**Figure 13:**
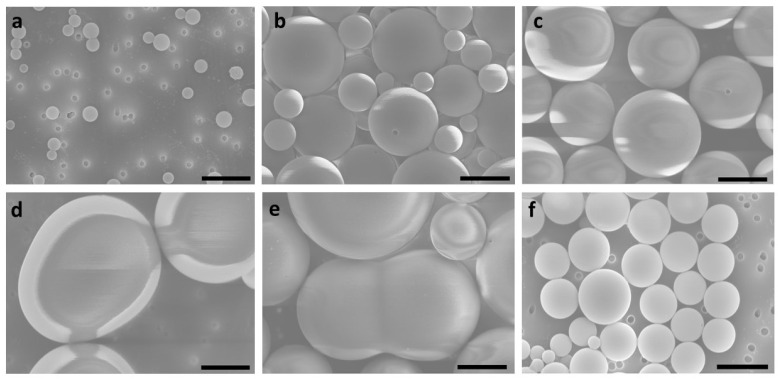
Exemplary SEM images to clarify the postulated ripening processes and behaviors of the PS-b-PAA vesicles in flow. (**a**) 400 rpm, 3 h, small vesicles; (**b**) 0 rpm, 24 h, large vesicles; (**c**–**e**) 100 rpm, 24 h, large vesicles (partially already deformed), as well as the incipient fission of vesicles; (**f**) 400 rpm, 24 h. Again, slightly smaller vesicles after the fission process. The scale bar is 1 µm.

These assumptions are supported by the SEM images (compare Figure 13), whereby no classical formation mechanism (morphology change over time) is shown here, but rather ripening processes derived from time and mixing intensity. It is postulated that there is a significant increase in vesicle size, mainly due to the fusion of vesicles with each other, and between the micelles and vesicles. This growth will continue until the kinetic equilibrium size is reached. However, if the vesicles are in a stream, the vesicles will grow to a critical size, and then deform and split due to the flow forces.

## 4. Conclusions

In this study, we investigated the influences of the process engineering aspects on structure formation, as well as studied the transition pathways from micelle to vesicle, in the self-assembled mesoscopic block copolymer structures of PS-b-PAA.

Specifically, the process parameters were the rate of addition of the selective solvent, the duration of the structure formation, and the mixing intensity. Our results demonstrate that a slower addition of the selective solvent to the polymer solution tends to result in more homogeneous systems, and consequently, narrower vesicle size distributions. In addition, the mixing intensity and the duration of the process have been demonstrated to have a significant effect on the morphologies and the dimensions of the polymer structures. We postulated that higher structure formation times and stirring speeds, and thus, higher shear rates, increase the collision probabilities between polymer structures, and thus shorten the time of phase transformations, which is further supported by CFD simulations.

Finally, from these results, formation mechanisms from micelles to vesicles could be derived, which are in good agreement with the previous findings in the literature, and furthermore complement them in a useful way. However, a classical formation mechanism (a change in morphology only over time) is not presented here, since both the time factor and the mixing intensity factor were investigated. Based on the observations made in this study, it can be assumed that a morphological transition occurs via the formation of micelles, which then assemble into “pearl necklace” structures and subsequently fuse or reorganize into smooth cylindrical structures. These cylinders then grow to a critical length to ultimately close into vesicles. The final step likely occurs via intermediate disk-like structures, forming the curvature of the disks into basket structures, and finally, the closure of the structure to form the final vesicle structure. These vesicles then ripen into larger structures through fusion processes. However, in a system where mechanical energy is continuously introduced (as in this case, through stirring), the vesicles can only grow to a critical size, as flow-induced forces cause deformation and trigger fission. Accordingly, the vesicle size is, on the one hand, determined by kinetics, but it is also significantly dependent on the process parameters.

## Figures and Tables

**Table 1:** Material data of the polystyrene-block-polyacrylic acid copolymer used.

	PS *	PAA *	PS-b-PAA
**M_n_ [g/mol]**	27,617	1761	29,378
**PDI**			≤1.1

* Molecular weights for the PS and the PAA moieties, calculated from the total molecular weight using the specific proportions of the blocks obtained from the manufacturer via the batch number.

**Table 2:** Influence of the rate of water addition on the average vesicle size and polydispersity of the polymer vesicles.

Water Addition Rate [µL/min]	Average Vesicle Size [nm]	Polydispersity
10	248	0.5
25	272	0.9
50	265	1.3
100	255	1.3
569	388	1.7

## Data Availability

Data are contained within the article or Supplementary Materials.

## Supplementary Materials

The following supporting information can be downloaded at: https://www.mdpi.com/article/10.3390/polym15071695/s1, Figure S1: Representation of the simulated velocity profile at stirring rates of 100 and 400 rpm. The arrows indicate the direction of the flow and the color the intensity of the fluid flow. All cells with a fraction of solution above 50% are considered.
