# Supplementary material for: Influence of Process Parameters on the Kinetics of the Micelle-to-Vesicle Transition and Ripening of Polystyrene-Block-Polyacrylic Acid
Source: Polymers (Basel). 2023 Mar 29;15(7):1695. doi: 10.3390/polym15071695 (PMC10096835; doi:10.3390/polym15071695)
Supplement: Supplementary file 1 [file polymers-15-01695-s001.zip › polymers-2218690-supplementary.pdf]

Supplementary Materials

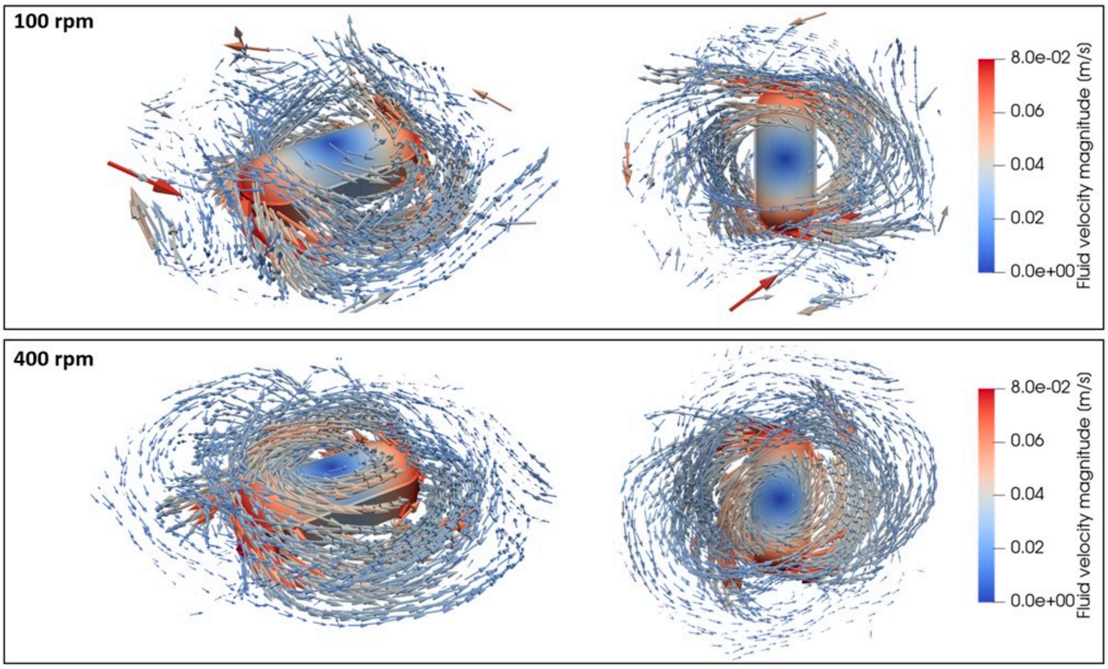

Figure S1. Representation of the simulated velocity profile at stirring rates of 100 and 400 rpm. The arrows indicate the direction of the flow and the color the intensity of the fluid flow. All cells with a fraction of solution above 50% are considered.
